# A Comparative Review of Demographics, Incidence, and Epidemiology of Histologically Confirmed Intracranial Tumors in Brazil and Bulgaria

**DOI:** 10.7759/cureus.2203

**Published:** 2018-02-19

**Authors:** George S Stoyanov, Jonathan S Sarraf, Boyko K Matev, Deyan L Dzhenkov, Martina Kitanova, Bogomil Iliev, Peter Ghenev, Anton B Tonchev, Yavor Enchev, Fernando Adami, Luis Eduardo W De Carvalho

**Affiliations:** 1 Department of General and Clinical Pathology, Forensic Medicine and Deontology, Medical University – Varna "prof. Dr. Paraskev Stoyanov", Varna, Bulgaria; 2 Genetic and Molecular Biology, Universidade Federal Do Pará, Belém, Pará, Brazil; 3 Student, Faculty of Medicine, Medical University – Varna “Prof. Dr. Paraskev Stoyanov”, Varna, Bulgaria; 4 Department of General and Clinical Pathology, Forensic Medicine and Deontology, Faculty of Medicine, Medical University – Varna “Prof. Dr. Paraskev Stoyanov”, Varna, Bulgaria; 5 Department of Neurosurgery and Ent Diseases, Division of Neurosurgery, Faculty of Medicine, Medical University – Varna “Prof. Dr. Paraskev Stoyanov”, Varna, Bulgaria; 6 Department of Anatomy and Cell Biology, Faculty of Medicine, Medical University – Varna “Prof. Dr. Paraskev Stoyanov”, Varna, Bulgaria; 7 Laboratory of Epidemiology and Data Analysis, Faculdadede Medicina Do Abc, Santo André, São Paulo, Brazil

**Keywords:** primary intracranial tumors, metastatic intracranial tumors, incidence, frequency, ethnic factors, demographics, pathology, oncology, statistics, comparison

## Abstract

Intracranial tumors (ICTs) attract numerous scientific teams and tremendous financial resources worldwide. These lesions of the central nervous system (CNS) can be both benign and malignant in biological behavior as well as local or metastatic in origin. We compared data from two studies on primary and metastatic ICTs from Brazil and Bulgaria, based on histopathologically confirmed ICTs from tertiary health centers. Primary ICTs significantly outweigh the frequency of metastatic ICTs. Primary ICTs represent 86.45% in Brazil and 69.17% in Bulgaria, with around 60% of their totals being malignant. There is a statistical dominance of tumors from the neuroepithelial origin, with the most common entry being glioblastoma multiforme. The second-most common primary ICT group comprises tumors of meningeal origin. Metastatic ICTs show great variance; 13.55% in Brazil and 31.38% in Bulgaria of all ICT cases being attributed to them. However, metastatic ICTs are even a more diverse group than neuroepithelial tumors, with the majority of this group comprising metastatic colorectal adenocarcinoma (almost exclusively in males), metastatic breast adenocarcinoma in females, metastatic pulmonary carcinomas (primarily from the non-small cell group with a male predominance), and metastatic melanoma with an even gender ratio.

## Introduction and background

Nowadays the scientific research of intracranial tumors (ICTs) attracts numerous scientific teams and tremendous financial resources worldwide [1-3]. These lesions of the central nervous system (CNS) and structures located in the cranial cavity can be both benign and malignant in biological behavior as well as local or metastatic in origin for malignant lesions [4-6]. They are rarely diagnosed in their early stages of biological development, require highly trained multidisciplinary medical team for their diagnosis and treatment, have a high impact on the patients and their relative quality of life, with some primary and metastatic entries having a very dismal survival prognosis, together with a high resource impact on the medical system [7-16].

Direct comparisons of primary and metastatic ICTs are very rare as they require a complex methodology of case gathering and calculation. Due to these exact reasons, comparative studies of primary and metastasic ICTs have not been carried out since the mid-1980s [17-19]. Since then, the standard has been that studies of primary CNS tumors must always apply to pathological criteria, while metastatic ICTs may only adhere to the radiological findings [20-32].

The aim of this review is to discuss our experiences and compare the findings regarding ICTs in our distinct populations, based on our data from Brazil, published by de Carvalho, et al. spanning across 17 years and 949 cases and from Bulgaria, published by Stoyanov, et al. spanning across five years and 798 cases [33-34].

## Review

The data was compared on the merit of major histopathological groups to accommodate the differences in the 2006 and 2016 World Health Organization (WHO) classification of tumors of the CNS intracranial tumors used in the two separate studies (Table 1) [6, 33-35]. Metastatic tumors were considered those not arising from the cranial cavity and the structures inside it, including infiltrative tumors such as lymphomas and leukemias. Lymphomas were compared based on their location of origin and systemic presentation, to conclude their origin as either primary CNS lymphoma or infiltrative CNS lymphomas.

**Table 1:** Comparison between ICTs in Brazil and Bulgaria with regards to gender, main histopathological group, age groups and median age ICT: intracranial tumor

Variable	Brazil		Bulgaria	
	N	%	N	%
Gender				
Male	383	48.06	558	69.92
Female	414	51.94	240	30.08
Histological type				
Tumors of Neuroepithelial tissue	326	40.90	248	31.08
Tumours of the meninges	172	21.28	213	26.69
Tumors of cranial and paraspinal nerves	50	6.27	12	1.51
Lymphomas and haematopoietic neoplasms	10	1.25	7	0.88
Germ cell tumors	9	1.13	8	1
Tumors of the sellar region	80	10.04	46	5.76
Metastatic tumours	108	13.55	254	31.83
Others	42	5.27	10	1.25
Total	797	100	798	100
Age group				
<20 years	199	24.96	148	18.46
20-39 years	190	23.81	106	13.28
40-59 years	254	31.86	345	43.23
>60 years	154	19.33	199	24.94
Median age	40		50	

Due to the different approaches, data gathering and WHO classifications used in the separate studies, 797 cases from a total of 1027 were used from the Brazilian population and 798 cases from a total of 822 were used from the Bulgarian population, accounting for a total of 1,595 ICT cases from both populations (Table 1). Both studies were based on collection of histopathologically confirmed specimens from tertiary health centers.

**Figure 1:**
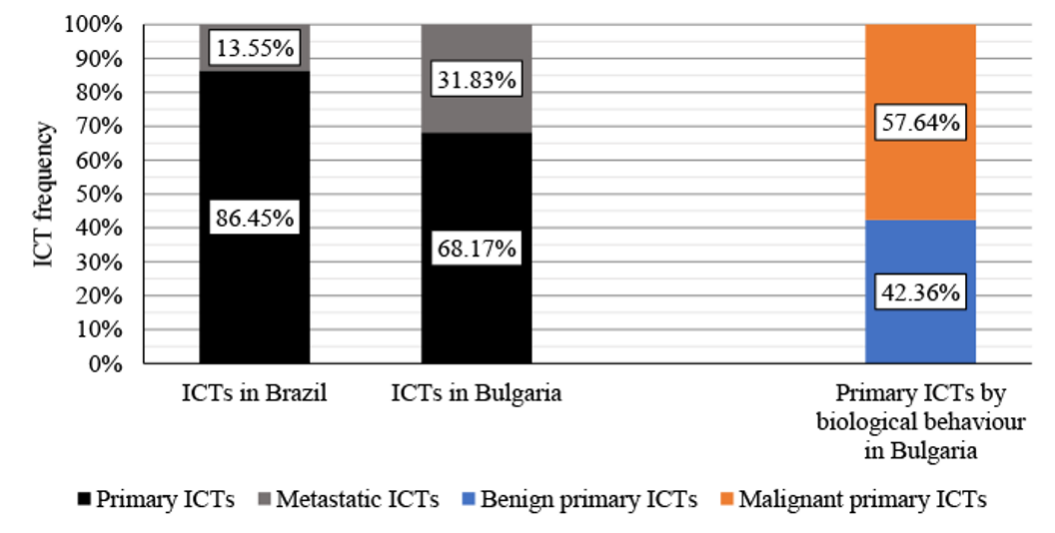
Comparison between ICTs in Brazil in Bulgaria, with benign and malignant comparison for primary ICTs in Bulgaria ICT: intracranial tumor

In both cohorts, primary ICTs significantly outweigh the frequency of metastatic ICTs [33-34]. Primary ICTs represent 86.45% in Brazil and 69.17% in Bulgaria of all ICTs, based on the histological verification criteria, with around 60% their total being malignant in nature (Figure 1).

Both studies found a statistical dominance of tumors from the neuroepithelial origin, with the most common entries being glioblastoma multiforme, with other astrocytic and glial tumors accommodating for a relatively small number of cases [33-34]. These results confirm other large case number reports [24-26].

The second most common primary ICT group is that of the tumors of meningeal origin, which are close to the total frequency of the very diverse group of neuroepithelial tumors (Table 1). However, since the major entry in this group are meningiomas, with some vascular and other rare entries accounting for a very small number of cases, meningiomas are statistically the most common ICT tumor type [33-34].

ICT groups such as primary lymphomas of the CNS, germ cell tumors and nerve sheath tumors account for a total of around 10% of all histologically confirmed cases of ICTs (Table 1). Tumors of the sellar region, despite being very common, clinically account for between five and 10% of all histologically confirmed cases of ICTs.

Metastatic ICTs are another big group, showing great variance in the two populations with 13.55% in Brazil and 31.38% in Bulgaria of all ICT cases being attributed to them (Table 1). However, metastatic ICTs are even a more diverse group than neuroepithelial tumors, with the majority of this group being due to metastatic colorectal adenocarcinoma (diagnosed almost exclusively in males), metastatic breast adenocarcinoma (diagnosed in females), metastatic pulmonary carcinomas primarily from the non-small cell group with a male predominance and metastatic melanoma with an even male to female ratio [33-34]. Some of the differences in the group of metastatic ICTs may be attributed to the variance of mutations described in the populations [36].

Other extremely rare entries such as pineal tumors, myofibroblastic tumors, and others can contribute to an aggregated total of five percent of histologically confirmed ICTs (Table 1).

Regarding metastatic location in the Bulgarian population, only 12.20% of cases of metastatic ICTs were found in the subtentorial space. There were no metastatic ICTs with predominant subtentorial metastatic space.

Despite the similarities of the histological findings, there are some visible differences in the results; in Brazil, the most affected age group is 40-59 years, followed by 0-19 years, whilst in Bulgaria, the most affected age group is 40-59 years, followed by >60 years (Table 1). These differences may be attributed to the significantly higher number of metastatic ICTs in the Bulgarian population when compared to the Brazilian population. Despite this, both studies describe a major peak of incidence in the age group 40-59 years when compared to the other age groups [33-34].

Despite the complexity of the separate groups of ICTs and the ethnic differences of both populations, ICTs remain an underestimated group of tumors with primary ICTs accounting for more than 2% of all newly diagnosed malignancies per year [37]. When adding the data of non-malignant primary ICTs and metastatic ICTs, the total incidence increases to around 4% of all cancer cases, equal to that of cancer types with which the population and general medical peers are much better acquainted, such as head and neck malignancies [38].

According to data collected from the Bulgarian population, the annual incidence of ICTs is 8.85 people per 100,000, comprised of 6.03 per 100,000 for primary ICTs and 2.82 for metastatic ICTs [34]. Furthermore, in other European populations, the reported incidence is even higher than the reported figures in our studies [3].

Although significantly more common than primary ICTs, metastatic ICTs represent only a small part of surgical specimens, as most patients with metastatic ICTs are either inoperable, suitable for radiosurgery only, or otherwise considered terminal.

The problematic of systemic analyses of ICTs demand a very strict choice of methods, as the different entries require different approaches to diagnosis. Although all tumors should be verified pathohistologically for the diagnosis to be certain, in the case of metastatic ICTs, this is seldom the case as they are often only diagnosed on clinical or radiological data alone. This choice of clinical methodological approach does not certify the tumor type, as the chance of a distant malignant entry, albeit often metastasizing to the CNS, does not exclude the presence of synchronous CNS tumor [31, 39].

Such cases are however very rare and most commonly presented only as a case report and case series and individual approaches are more often than not taken as absolute. This is due to combined pathohistological and radiological criteria that would severely narrow down the study cohort and almost always report the same findings as the pathological criteria alone, while any cases that would be proven otherwise by pathology would only fit in the statistical error part of a radiological study. A further option on the matter is the rarely undertaken autopsy finding study as it reports on a very small series of cases, even in a greater timeframe [40].

Despite differences in some aspects of the individual study designs, the reported results are quite similar and a further representation of the adequacy of the individually given data. If taken together, the data volume spans across a total of 22 calendar years and 1595 cases and is the most complete representation of primary and malignant tumors of the CNS up to date, outweighing similar studies carried out in the past, by a great number of cases [17-18].

Furthermore, both studies report a greater number of cases and further increase the statistical understanding and epidemiological data of CNS tumors such as de Carvalho, et al. which also depicts CNS spinal tumors, whilst Stoyanov, et al. depicts non-tumor volume-occupying lesions such as cysts and abscesses with a total annual incidence of 0.27 people per 100,000.

As seen in a direct comparison of the individual data, the findings are similar in a number of categories, despite some visible differences, primarily in median age and total male to female ratio. Still, the findings are not that dissimilar when compared to individual reports on primary or metastatic ICTs alone [20-21, 24-26]. This is both due to the pathological criteria of both studies and the large number of patients reported in each study.

One of the most intriguing parts of the comparison of results is the wide similarity of the results, despite the significant ethnic differences of the populations, questioning the impact of ethnic factors in CNS tumors pathobiology as described in previous studies [41-42].

## Conclusions

Although significantly more common than primary ICTs, metastatic ICTs represent only a small part of surgical and histopathological specimens, as most patients with metastasic ICTs are either inoperable, suitable for radiosurgery only, or otherwise considered terminal.

The demographic data represented in both studies demonstrate a severe difference in age groups on the background of the similar histological findings, which may be the only ethnic aspect confirmed by the reports, this time confirming the demographic specifics of ICTs.

Despite all the individually reported results and the lesser percentage of metastatic ICTs, both sets of authors agree that the pathological verification criteria do not report the total incidence of metastatic ICTs, which should, in total, given the globally increasing reported incidence of cancer, outweigh the percentage of primary ICTs.
